# Clinical observation of phacoemulsification under the low perfusion pattern and low negative pressure in patients with low corneal endothelial cell density

**DOI:** 10.1186/s12886-023-03095-w

**Published:** 2023-07-31

**Authors:** Yan Lin, Biting Zhou, Yihua Yao, Qin Ye, Yihua Zhu, Xiaohui Wang

**Affiliations:** Department of Ophthalmology, The First Affiliated Hospital of Fujian Medical University, Fuzhou, China

**Keywords:** Cataract, Phacoemulsification, Corneal endothelial cell, Low perfusion pattern

## Abstract

**Background:**

To observe the safety and effect of phacoemulsification combined with intraocular lens (IOL) implantation in patients with low corneal endothelial cell density (CD) under the low perfusion pattern with low negative pressure.

**Methods:**

In this retrospective case series study, a total of 16 patients (17 eyes) were studied. They had all been diagnosed with low corneal endothelial (CD) and cataracts in the First Affiliated Hospital of Fujian Medical University from December 2019 to October 2021. They underwent phacoemulsification combined with IOL implantation under the low perfusion pattern with low negative pressure. The variations of corneal endothelial( CD), coefficient of variation (CV) of the cell area, central corneal thickness (CCT), visual acuity, and intraocular pressure before and after the operation were observed and assessed. Then a paired *t-*test, repeated measures analysis of variance, and Pearson correlation analysis were adopted for data analysis.

**Results:**

The mean intraocular pressure of the 17 eyes was 16.88 ± 6.47 mmHg before the operation and 14.41 ± 3.10 mmHg after the operation, showing a statistically significant difference of *t* = 2.222, and *p* = 0.041. Before the operation, the mean visual acuity was 0.16 ± 0.09, and after the operation, it was 0.45 ± 0.16, displaying a statistically significant difference of *t* = -9.917, *p* < 0.001. Before and after the operation, four of the 17 eyes had no detectable CD. The mean CD of the other 13 eyes at one month after the operation (644.308 ± 106.24 cells/mm^2^) was lower than that before the operation (709.62 ± 119.19 cells/mm^2^), and the differences were statistically significant (F = 20.044, *p* < 0.001). However, no statistically significant differences were found in the mean CV before the operation (31.23 ± 4.21), and at one month after the operation (32.62 ± 3.80; F = 2.130, *p* = 0.157). Moreover, the mean CCT of 14 eyes at one month after the operation (562.72 ± 27.82 μm) was larger than that before the operation (534.79 ± 24.69 μm).

**Conclusions:**

The low perfusion pattern with low negative pressure is safe and effective for corneal endothelial dysfunction patients complicated with cataracts.

## Background

Cataracts can mainly be treated by extraction combined with intraocular lens (IOL) implantation. At present, phacoemulsification, as a cataracts extraction method, has been widely applied in clinics because it causes small wounds and a low postoperative astigmatism rate, with faster visual acuity rehabilitation [1]. However, the ultrasound energy, the sheer force generated by the vortex of perfusion fluid of the anterior chamber, and the floating lens debris in the anterior chamber will damage corneal endothelial cells to some extent, which will reduce the corneal endothelial cell density (CD). Hence, cataracts extraction combined with IOL implantation is not particularly safe for some patients with corneal endothelial injury [2]. There are 1400–2500/mm^2^ corneal endothelial cells in normal adults. Clinically, some patients with low corneal endothelial CD combined with cataracts are often intractable cases. Therefore, the way to protect the corneal endothelium during the operation is still a focus of cataracts surgeons [3]. In the present study, the clinical effect of cataracts extraction in 17 eyes with corneal endothelial CD < 1,000/mm^2^ complicated by cataracts was retrospectively assessed, and the application effectiveness of the low perfusion pattern with low negative pressure in such patients was explored.

## Methods

### Research subjects

This study protocol adhered to the principle stated in the Declaration of Helsinki and was approved by the Ethics Committee of the First Affiliated Hospital of Fujian Medical University. A total of 16 patients (17 eyes) with a preoperative corneal endothelial CD less than 1000/mm^2^ (554/mm^2^–996/mm^2^) who received phacoemulsification combined with IOL implantation in the First Affiliated Hospital of Fujian Medical University from December 2019 to October 2021 were selected. Based on the Emery nuclear grading system, the lens nuclei of all eyes were classified into grades III–IV before the operation. There were 12 eyes with grade III nuclei and 5 with grade IV nuclei. Exclusion criteria were set as follows: (1) patients with definite retinal or optic nerve diseases or other eye diseases, (2) those who have had other operations performed or have suffered from external injuries in the operated eyes, (3) those with secondary glaucoma or severe uveitis after the operation, and (4) those with preoperative corneal edema.

### Operation methods

One ophthalmologist carried out all operations. There were 17 eyes with grades III–IV nuclei. A Stellaris phacoemulsification machine (Bausch + Lomb, USA) was employed for cataract phacoemulsification under the continuous ultrasound energy release mode. During the phacoemulsification, an appropriate amount of 3% sodium hyaluronate was first injected into the anterior chamber to form the anterior chamber operating space using the “soft-shell technique” until the injection fluid was uniformly attached to the corneal endothelial surface. The phacoemulsification was performed under the low perfusion pattern (height of perfusion bottles of 95 cm) with low negative pressure (< 250 mmHg), The energy output of the ultrasonic device is characterized by three modes: continuous, pulse, and burst. In this study, we opted for the burst mode, with the maximum energy set at 60%, the maximum vacuum pressure ranging from 250 to 300 mm Hg, and a flow rate of 40ml/min. The single burst blockage duration was configured at 50ms, with the foot pedal employed for linear control of the number of burst blockages. Beginning at the third gear, a burst occurred every 2.5s, while continuous burst blockages were executed at the lowest point of the third gear.

Next, the lens nucleus was removed through phacoemulsification using the soft-shell technique, while residual cortex was removed through automated irrigation and aspiration. Subsequently, 3% sodium hyaluronate was injected into the anterior chamber again to support the capsular bag, Utilizing the protection afforded by the soft-shell technique on the endothelium, we processed the nuclear fragments using the split-flip method. The ultrasonic probe, which utilizes linear ultrasonic energy, was inserted directly into the center of the lens nucleus, thereby securing it. An auxiliary incision was used to insert the splitter into the equator of the lens nucleus. Relative movement of the splitter and the ultrasonic probe led to misplacement at the point of contact, thereby splitting the lens nucleus into two halves. The lens nucleus was then rotated 90° and progressively cut into smaller pieces using the same methodology. These nuclear fragments were aspirated using vacuum suction and subsequently flipped out from within the lens capsule. an IOL was implanted, and the viscoelastic agent was removed to form a watertight anterior chamber.

### Clinical outcome measurements

Routine visual acuity and intraocular pressure (IOP) were conducted a non-contact tonometer and slit lamp examination were performed before and after the operation at 1 day and 1month respectively. The central corneal thickness (CCT), corneal endothelial CD, and coefficient of variation (CV) of the cell area were measured using specular microscope (TOPCON, Japan) by a trained ophthalmologic physician. The loss rate of endothelial cells was calculated as follows: corneal endothelial cell loss rate = (preoperative CD-postoperative CD)/preoperative CD×100%.

### Statistical methods

This was a retrospective case series study. SPSS 21.0 was adopted for statistical analysis. Data were shown as mean ± SD (x̅±s). Paired *t*-test was used to investigate the differences in the IOP and visual acuity before and after the operation. The differences in CV, CD and CCT were analyzed using repeated measures analysis of variance (RM ANOVA). In addition, a Pearson correlation analysis was conducted to analyze the correlations of each factor with the corneal endothelial cell loss rate and postoperative CCT. *p* < 0.05 represented that the difference was statistically significant.

## Results

### Demographic and ocular characteristics of patients

Seventeen eyes of 16 cataract patients with low endothelial CD underwent phacoemulsification and IOL implantation, including 7 eyes of 7 males and 10 eyes of 9 females. The average age of the patients was 68.9 ± 5.25 years. The causes of poor corneal endothelial CD in these patients including glaucoma, keratitis, Fuchs endothelial dystrophy, iridoschisis and some unknown reasons. The average energy of superemulsion is 11 ± 4%. The average time of superemulsion is 27 ± 11 s, the cumulative energy is 325 ± 215%.Among the 17 eyes, corneal endothelial CD could not be measured in four eyes. Meanwhile, three eyes of them had no detectable CCT. Thus, corneal endothelial CD and CV of 13 eyes and CCT of 14 eyes were analyzed in this study (Tables 1 and 2).

**Table 1:** Preoperative information of patients

Patient	Age/Sex	Type	IOP (mmHg)	Visual acuity	CD (cells/mm^2^)	CV	CCT (µm)
1	74/M	Unknown	14	4.3	803	31	534
2	74/F	Unknown	15	4.2	762	24	518
3	69/F	Glaucoma	22	4.1	655	29	589
4	71/F	Fuchs	11	4.5	-	-	-
5 (OD)	67/F	Fuchs	11	4.3	621	36	559
5 (OS)	67/F	Fuchs	12	4.5	563	31	526
6	68/M	Keratitis	14	4.5	833	35	562
7	65/M	Keratitis	15	3.7	-	-	-
8	70/M	Glaucoma	24	4.1	723	27	537
9	72/M	Keratitis	12	4.0	689	32	517
10	62/F	Iridoschisis	13	4.4	594	35	547
11	66/M	Glaucoma	27	4.1	791	28	521
12	68/F	Glaucoma	17	4.4	-	-	-
13	81/M	Keratitis	8	4.0	-	-	503
14	58/F	Glaucoma	26	3.3	554	27	554
15	73/F	Glaucoma	16	4.0	672	37	514
16	65/F	Glaucoma	30	4.1	965	35	506

**Table 2:** The postoperative outcomes of patients after one day (1D) and one month (1 M)

Patient	IOP (mmHg)	VA	CD (cells/mm^2^)		Loss rate (%)		CV		CCT (µm)	
			1D	1 M	1D	1 M	1D	1 M	1D	1 M
1	13	4.7	756	740	5.85	7.85	35	34	599	550
2	17	4.8	747	752	1.97	1.31	25	27	528	520
3	16	4.6	621	640	5.19	2.29	30	32	603	600
4	14	4.8	-	-	-	-	-	-	-	-
5 (OD)	10	4.7	543	550	12.56	11.43	38	37	583	570
5 (OS)	13	4.7	521	518	7.46	7.99	29	33	552	540
6	15	4.8	671	721	19.45	13.45	33	36	630	580
7	12	4.6	-	-	-	-	-	-	-	-
8	18	4.4	680	711	5.95	1.66	23	26	650	621
9	15	4.6	632	642	8.27	6.82	34	31	540	530
10	14	4.9	512	520	13.80	12.46	34	36	580	569
11	20	4.5	631	600	20.23	24.15	26	30	621	583
12	11	4.6	-	-	-	-	-	-	-	-
13	10	4.5	-	-	-	-	-	-	610	554
14	19	4.1	511	503	7.76	9.21	28	29	589	571
15	11	4.7	645	630	4.02	6.25	41	36	578	553
16	17	4.7	892	849	7.56	12.02	39	37	556	537

### Comparisons of visual acuity and IOP before and after the operation

As shown in Table 3, the mean IOP was 16.88 ± 6.47 mmHg and 14.41 ± 3.10 mmHg pre- and postoperation, respectively, showing a statistically significant difference (*t* = 2.222, *p* = 0.041). In addition, the mean visual acuity was 4.15 ± 0.31 preoperation. After operation, the visual acuity increased to 4.63 ± 0.19 significantly (*t* = -9.908, *p* < 0.001). The IOP of all patients was within the normal range. During the long-term follow-up, no obvious abnormality appeared in the IOP of all patients.

**Table 3:** Comparisons of IOP and visual acuity before and after the operation ($\bar x \pm s$)

	IOP	Visual acuity
Before operation	16.88 ± 6.47	4.15 ± 0.31
After operation	14.41 ± 3.10	4.63 ± 0.19
*t*	2.222	-9.908
*p*	0.041	< 0.001

### Changes of corneal endothelial cells before and after the operation

As illustrated in Table 4, the mean CD at one day after the operation (643.23 ± 111.04 cells/mm^2^) and at one month after the operation (644.31 ± 106.24 cells/mm^2^) were lower than that before the operation (709.62 ± 119.19 cells/mm^2^), and the differences between preoperation and one month after operation were statistically significant (F = 21.234, *p* < 0.001). However, compared with preoperative CV (31.31 ± 4.11), no statistically significant difference was found in the CV values at one month postoperatively (32.62 ± 3.80; F = 2.130, *p* = 0.157). Moreover, the mean CCT at one day after the operation (587.07 ± 35.00 μm) and one month after the operation (562.71 ± 27.82 μm) were larger than that before the operation (534.79 ± 24.69 μm), with statistically significant differences (F = 27.953, *p* < 0.001). At one day after the operation, corneal stromal edema and wrinkling of a posterior elastic layer occurred in 5 eyes, all of which were from patients with grade IV nuclear cataracts. These phenomena gradually subsided 3–10 days after the operation. The cornea was transparent in the remaining patients, and there was no wrinkle in the posterior elastic layer. The CCT was larger than 600 μm in 2 eyes during the follow-up, but it was not detectable in 3 of the eyes. However, the cornea remained transparent for a long time during the follow-up, and there was no case of bullous keratopathy.

**Table 4:** Comparisons of CD, CV, and CCT before and after the operation ($\bar x \pm s$)

	CD	CV	CCT
Before operation	709.62 ± 119.19	31.31 ± 4.11	534.79 ± 24.69
One day after the operation	643.23 ± 111.04	31.92 ± 5.62	587.07 ± 35.00
One month after operation	644.31 ± 106.24	32.62 ± 3.80	562.71 ± 27.82
F	21.234	1.939	27.953
*p*	< 0.001	0.166	< 0.001

### Correlations of each factor with corneal endothelial cell loss rate and CCT after the operation

The loss rate of corneal endothelial cells after the operation had no significant correlations with CV before the operation. Besides, the CCT after the operation was not significantly related to CV before the operation. According to the correlation analysis, the CV before the operation was positively related to the CV at one day after the operation, with a correlation coefficient of 0.893, and it was positively correlated with CV at one month after the operation, with a correlation coefficient of 0.929.

## Discussion

The structural integrity and functional soundness of the corneal endothelial cell layer are crucial for maintaining the normal physiological function of the cornea. Decreased endothelial CD and endothelial pump dysfunction result in corneal endothelial edema, which seriously damages visual acuity [4]. Cataracts patients with the symptoms mentioned above are intractable. And they will be at the risk of blindness if not treated. In this study, patients with low corneal endothelial CD received cataract extraction combined with IOL implantation, and all of them gained functional visual acuity after the operation. Although their postoperative visual acuity might not return to the normal level, it could be remarkably improved and remain stable for a long time, which substantially improved the patients’ quality of life [5].

Both ultrasound energy and mechanical shear force can reduce endothelial cells in cataract extraction. According to the reports of previous studies, corneal endothelial cells are decreased by 4–25% on average after phacoemulsification combined with IOL implantation [6, 7]. Patients with low endothelial cells have a very high sensitivity to injuries [8]. A study by Ken Hayahihi et al. has shown that the difference in the loss rate of corneal endothelial cells is not statistically significant between patients receiving cataracts extraction and normal patients. The corneal edema rate is high after the operation, but it recovers 1 month after the operation [9]. A study by Yamazoe et al. [10] has revealed no significant difference in endothelial cell loss after phacoemulsification in patients with low corneal endothelial CD complicated by grade IV or below nuclear cataracts. However, extracapsular cataract extraction may be better for patients with grade V nuclear cataracts. In the present study, the nuclear cataracts of all the patients were in grades III–IV, so all of them underwent phacoemulsification.

As indicated by the relevant literature and clinical practice, endothelial cell injury after phacoemulsification mainly results from mechanical and thermal injuries [11]. The mechanical injury is primarily caused by the insertions and removal of phacoemulsification needles and the rotation of lens nuclear fragments in the eye, whereas the thermal injury is mainly associated with phacoemulsification energy [2, 12, 13]. Hence, phacoemulsification was performed under low perfusion with low negative pressure in this study, which prevented the injury of corneal endothelium from excessive phacoemulsification energy and improved the anterior chamber stability, thus contributing to the IOP control and the occurrence of the shallow anterior chamber [14]. Besides, the hardness of the lens nucleus was all in grades III–IV, and the phacoemulsification was performed in a short time period under low energy [3]. The short-time and low-energy phacoemulsification parameters and skillful operations minimized the injury of corneal endothelial cells [15]. Among 17 eyes, only 5 had corneal edema after the operation, and the edema obviously subsided one month after the operation. Although only 2 eyes had postoperative CCT exceeding 600 μm, corneal decompensation did not occur. The following advantages characterize the phacoemulsification technology with small parameters: (1) The anterior chamber is slightly disturbed, so the incidence rates of surge phenomenon, shallow anterior chamber, and vitreous prolapse during operation can be reduced. (2) The suction of viscoelastic agents is slowed down to effectively protect the corneal endothelium. (3) The lens nucleus is slowly smashed to increase the operation’s safety. (4) Low perfusion pressure can limit the increase of IOP during phacoemulsification and avoid the injury of the corneal endothelium caused by high IOP during operation.

Effective surgical techniques were adopted to protect endothelial cells against injury to the largest extent during operation [16]. The loss rate of endothelial cells was 8.99% in patients with a small corneal endothelial cell count, which could be attributed to the low perfusion pattern with low negative pressure adopted during operation. This result is similar to the study results of Doors and Chen; that is, the higher the total ultrasound energy and the longer the ultrasound time, the more serious the corneal endothelial cell injury [17, 18].

Previous literature has reported that it is of great significance to evaluate corneal endothelial cells’ area, number, and morphology to determine the corneal endothelium’s health condition [19]. The injured corneal endothelial cells will experience death and there is no chance of recovery. The missing endothelial cells are repaired by the enlargement and migration of their surrounding corneal endothelial cells, so the endothelial cell area will become larger, and the normal hexagonal appearance will disappear after injury [20, 21]. CV is an index for endothelial deformation, whose sensitivity is higher than CD in reflecting endothelial dysfunction. The larger the CV, the more notable the inconsistency of the cell area. With poor endothelial function, patients with low corneal endothelial CD are more sensitive to injury, and their cells are larger in areas with higher heteromorphism [22]. In this study, CV had no statistically significant difference in patients with low corneal endothelial CD before and after the operation, indicating the effectiveness and significance of protecting the corneal endothelium during operation.

CCT is another index reflecting the functional state of the corneal endothelium. The corneal endothelium damaged during cataracts extraction will cause corneal edema and increase the corneal thickness [23, 24]. In the present study, the postoperative mean CCT was significantly increased compared with preoperation. Among 17 eyes, 2 eyes had CCT > 600 μm after the operation, but corneal endothelial dysfunction was found in none of them during the follow-up. The CD was not detectable before and after operation in 4 patients, with three eyes had undetectable CCT. However, phacoemulsification was performed since they had transparent corneas and grade III nuclear cataracts, and their corneas remained transparent after brief edema in the postoperative follow-up.

In this study, the reason corneal endothelial cells have low density but can tolerate phacoemulsification and IOL implantation is that, apart from a series of procedures taken to protect the endothelium during the operation, the apoptosis of corneal endothelial cells caused by chronic inflammation is a long process, in which the surrounding endothelial cells repeatedly deform, migrate, and fuse [25]. This process enhances the pump function and increases the expansion potential of endothelial cells. That is, the maximum healing reserve is gradually increased [26]. However, studies reveal that temporarily transparent corneas in patients may gradually develop corneal edema or even corneal decompensation 5 years after the operation. The limitations of this study include the small sample size and short follow-up time.

## Conclusions

This study demonstrates that phacoemulsification with low perfusion pattern and low negative pressure is safe and effective for patients suffering from cataract and corneal endothelial cell dysfunction. Further studies that containing more samples and long follow-up time are needed to confirm the findings.

## Data Availability

The data analyzed during the current study are available from the corresponding author on reasonable request.

## List of abbreviations

IOL: Intraocular lens
CD: Cell density
CV: Cell variation
CCT: Central corneal thickness
IOP: Intraocular pressure
RM ANOVA: Repeated measures analysis of variance
